# Iodine Status Assessment in South African Adults According to Spot Urinary Iodine Concentrations, Prediction Equations, and Measured 24-h Iodine Excretion

**DOI:** 10.3390/nu10060736

**Published:** 2018-06-07

**Authors:** Karen E. Charlton, Lisa J. Ware, Jeannine Baumgartner, Marike Cockeran, Aletta E. Schutte, Nirmala Naidoo, Paul Kowal

**Affiliations:** 1 School of Medicine, University of Wollongong, Wollongong 2500, New South Wales, Australia; 2 Illawarra Health and Medical Institute, University of Wollongong, Wollongong 2500, New South Wales, Australia; 3 Hypertension in Africa Research Team (HART), North-West University, Potchefstroom 2531, North West Province, South Africa; lisa.jayne.ware@gmail.com (L.J.W.); alta.schutte@nwu.ac.za (A.E.S.); 4 MRC/Wits Developmental Pathways for Health Research Unit, University of the Witwatersrand, Johannesburg 2193, Gauteng, South Africa; 5 Centre of Excellence for Nutrition (CEN), North-West University, Potchefstroom 2531, North West Province, South Africa; jeannine.baumgartner@nwu.ac.za; 6 Statistical Consultation Services, North-West University, 11 Hoffman Street, Potchefstroom; Private Bag X6001, Potchefstroom 2520, North West Province, South Africa; Marike.Cockeran@nwu.ac.za; 7 MRC Research Unit for Hypertension and Cardiovascular Disease, North-West University, Potchefstroom 2531, North West Province, South Africa; 8 World Health Organization (WHO), Avenue Appia 20, CH-1211 Geneva 27, Switzerland; naidoon@who.int (N.N.); kowalp@who.int (P.K.); 9 Research Centre for Generational Health and Ageing, University of Newcastle, Newcastle 2308, New South Wales, Australia

**Keywords:** iodine, median urinary concentration, 24 h urine collection, prediction equations, agreement, estimated average requirement

## Abstract

The iodine status of populations is conventionally assessed using spot urinary samples to obtain a median urinary iodine concentration (UIC) value, which is assessed against standard reference cut-offs. The assumption that spot UIC reflects daily iodine intake may be flawed because of high day-to-day variability and variable urinary volume outputs. This study aimed to compare iodine status in a sample of South African adults when determined by different approaches using a spot urine sample (median UIC (MUIC), predicted 24 h urinary iodine excretion (PrUIE) using different prediction equations) against measured 24 h urinary iodine excretion (mUIE). Both 24 h and spot urine samples were collected in a subsample of participants (*n* = 457; median age 55 year; range 18–90 year) in the World Health Organization Study on global AGEing and adult health (SAGE) Wave 2 in South Africa, in 2015. Kawasaki, Tanaka, and Mage equations were applied to assess PrUIE from predicted urinary creatinine (PrCr) and spot UIC values. Adequacy of iodine intake was assessed by comparing PrUIE and mUIE to the Estimated Average Requirement of 95 µg/day, while the MUIC cut-off was <100 µg/L. Bland Altman plots assessed the level of agreement between measured and predicted UIE. Median UIC (130 µg/L) indicated iodine sufficiency. The prediction equations had unacceptable bias for PrUIE compared to measured UIE. In a sample of adult South Africans, the use of spot UIC, presented as a group median value (MUIC) provided similar estimates of inadequate iodine status, overall, when compared to EAR assessed using measured 24 h iodine excretion (mUIE). Continued use of MUIC as a biomarker to assess the adequacy of population iodine intake appears warranted.

## 1. Introduction

Iodine deficiency is the largest preventable cause of brain damage and mental impairment worldwide. Populations that consume diets that contain small amounts of fish and seafood, moderate to low quantities of milk and dairy products, and include locally produced fruits and vegetables grown in iodine-poor soils are likely to be iodine deficient. For this reason, in order to prevent iodine deficiency disorders, the World Health Organization (WHO) recommends universal salt iodization (USI), where all salt for human and animal consumption is iodized [1].

Three quarters of the world’s population in 2016, in a total of 130 countries, was estimated to use iodized salt [2,3]. The 2016 global estimate of iodine nutrition, based on surveys of school-age children conducted between 2002 and 2016, showed that iodine intake is insufficient in 15 countries, sufficient in 102, and excessive in 10 countries [4,5]. This represents a halving of the number of countries with insufficient iodine intake over five years, from 32 in 2011 [6] to 15 countries in 2016 [4], and reflects continuing progress to improve the coverage of iodized salt at a national level.

Children born to women who are iodine deficient are at risk of impaired psychomotor development and behavioral problems [7]. Even mild iodine deficiency in pregnancy is associated with learning deficits in offspring at age 8–9 year [8], which persist through to adolescence, despite adequate iodine exposure during early childhood.

Monitoring and surveillance of iodine status is routinely conducted in many countries and reported against a global iodine scorecard [9]. Approximately 90% of dietary iodine is excreted in the urine, therefore, the most commonly used biomarker of iodine intake is urinary iodine concentration (UIC) in collections of casual or spot urine samples for the assessment of median UIC (MUIC) in a population group [10]. This is the method recommended by the WHO/ICCIDD (International Council for Control of Iodine Deficiency Disorders), with iodine sufficiency indicated if the MUIC for a non-pregnant population exceeds 100 µg/L, and if no more than 20% of the population have a urinary iodine concentration below 50 µg/L [1]. The iodine scorecard, published periodically by the Iodine Global network, collates country-level data of MUIC in both school-age children and in women of a reproductive age. Adequate iodine intake in school-age children corresponds to median UIC values in the range of 100–299 µg/L, while for pregnant women the range indicating adequacy is 150–249 µg/L [1]. In the absence of recent national surveys, sub-national UIC surveys are included in the scorecard, but those data should be interpreted with caution [6].

The recommended method of assessing success or failure of fortification programmes in correcting iodine deficiency, while avoiding excess, is determined by assessing MUIC every five years in school-aged children (6–12 year). This indicator is also included in the battery of measures used to capture various aspects of food insecurity, as recommended by the Committee on World Food Security (CFS) [11]. The choice of indicators, including for iodine, was based on both expert judgment and the availability of data with sufficient coverage to enable comparisons across regions and over time. Under the food security pillar of “utilisation”, nutritional indicators include: The prevalence of inadequate iodine intakes using MUIC, along with the prevalence of stunting, wasting, and underweight in children; underweight in adults; anaemia in pregnancy and children; population-level vitamin A deficiency; and improved access to sanitation and clean water.

Use of MUIC, as a measure of population-level iodine status, is based on the assumption that daily urinary excretion of iodine closely reflects iodine intake in non-pregnant populations. The other assumption is that a spot urine collection reflects urinary excretion over the entire day [9]. It is well documented that UIC should not be used to assess iodine status in individuals because of its high intra- and inter-individual variation [12,13,14,15]. A 13-month longitudinal study of 16 healthy men living in an area of mild to moderate iodine deficiency has provided information on the number of spot urine samples needed to estimate the iodine level in a population. To obtain a MUIC with 95% confidence within a precision range of either 10% or 5%, urine samples are needed from 125 and 500 individuals, respectively. For an individual, to obtain an iodine value within a precision range of +/− 20%, 12 or more repeated spot urine samples are required [16]. Other authors have demonstrated that 10 repeat collections are required for urinary iodine from either spot samples or 24-h samples to provide a reliable estimate of individual iodine status in women [13].

The assumption that MUIC reflects daily iodine intake may be flawed because of high day-to-day variability and variable urinary volume outputs. The aim of this study was to compare iodine status in a sample of South African adults, recruited countrywide, when determined by different approaches using a spot urine sample (median UIC (MUIC) and predicted 24 h urinary iodine excretion (PrUIE) using different prediction equations) compared to measured 24 h urinary iodine excretion (mUIE).

## 2. Materials and Methods

The study sample is from a nested tobacco and salt sub-study included in the World Health Organization Study on global AGEing and adult health (WHO SAGE) [17]. WHO SAGE is a multinational cohort study examining the health and wellbeing of adult populations and the ageing process. Two waves of this longitudinal study have been completed in China, Ghana, India, Mexico, Russia, and South Africa [18]. In total, 42,464 respondents were recruited across the six countries for Wave 1 (2007–2010), including 4223 respondents in South Africa (9% 18–49 years; 40% 50–59 years; 51% 60+ years). Respondents were recruited from selected probability sampled enumeration areas (EAs) using a multi-stage cluster sampling strategy, with stratification by province, residence, and race. Urine capture was included as part of the SAGE South Africa Wave 2 data collection. The Wave 2 data collection sampling strategy in South Africa (2015) was designed to follow up on Wave 1 households where possible, accounting for attrition with systematic random sampling of new households. This process uses EA aerial photographic maps on which dwellings are clearly visible and, starting at a random point on the periphery of the EA, follows pre-determined routes.

During Wave 2 data collection, 20 survey teams (one nurse and three interviewers per team) simultaneously collected data and samples from respondents across all provinces in the country over a five-month period. Respondents that were recruited to provide urine samples (*n* = 1200) were from the first households visited within each EA, as a means to simplify logistics and reduce sample transit time to the central Durban laboratory. Inclusion criteria for urine collection were: Respondent must be part of the WHO SAGE cohort, with no indication of urinary incontinence or any other condition that could impede 24-h urine collection; and, if female, not menstruating, pregnant, or breastfeeding on the day of collection.

All survey teams were trained with support from the WHO Geneva. As part of the larger survey, anthropometry, household and individual questionnaires, blood sampling, blood pressure (BP), and physical function tests were completed, as described previously in SAGE Wave 1 [18]. Interviewers spoke the respondents’ home languages, with consent forms available in the most widely spoken languages for each area. All respondents provided written informed consent prior to taking part in the study. The study complies with the ethical principles for medical research involving human subjects as per the Declaration of Helsinki [19]. The WHO Ethics Review Committee approved the study [RPC149]. Local ethical approval was obtained from the North-West University Human Research Ethics Committee (Potchefstroom, South Africa), and the University of the Witwatersrand Human Research Ethics Committee (Johannesburg, South Africa).

### 2.1. Urine collection

The protocol used for collection of the 24-h urine samples followed the WHO/PAHO (Pan American Health Organization) guidelines [20]. Respondents were requested to collect all urine produced over 24 h, excluding the first pass of urine on day 1, but including the first urine of the following morning (day 2), in a 5-L plastic container, with 1 g thymol as a preservative. The spot sample was collected without preservative from the second urine passed on day 1 (marking the start of the 24-h collection) and decanted into three 15 ml Porvair tubes (Porvair Sciences, Leatherhead, UK and kept in a cool box powered by the fieldwork vehicles. The 24-h sample volumes were recorded upon collection the next morning and any aliquots generated thereafter (4 × Porvair tubes), with all samples then shipped to the laboratory maintaining the cold chain. Thymol, a crystalline natural derivative of the Thyme plant, has been shown to prevent changes in urinary creatinine, sodium, and potassium concentrations for up to five days [21]. Even though there is no evidence that the addition of preserving substances, such as thymol, affect urinary iodine concentrations [22], we undertook testing to examine the influence of adding thymol or HCl to urine samples (*n* = 20) on urinary iodine concentrations. The results indicated no significant or relevant (below assay coefficient of variation) differences when compared to samples without added preservatives (results not shown here). Incomplete 24-h urine collections were assumed if: Total volume ≤300 mL; or creatinine excretion ≤4 mmol/day (women) or ≤6 mmol/day (men) [23].

### 2.2. Urine Analysis

Samples from spot and 24 h urine for iodine analysis were stored at −20 °C and batch analysed using the Sandell-Kolthoff method with ammonium persulfate digestion and microplate reading [24] at the North-West University Centre of Excellence for Nutrition. The spot and 24 h urine samples from a single participant were analysed within the same assay to exclude inter-assay variation. The laboratory participates successfully in the Program to Ensure the Quality of Urinary Iodine Procedures (EQUIP, U.S. Centres for Disease Control and Prevention, Atlanta GA, USA) [25], and internal quality control samples (two different levels) were analysed with each assay.

### 2.3. Comparison of Measured UIE with MUIC and Predicted UIE Using Spot Iodine Concentrations

An electronic data capture system was used during face-to-face interviews. SPSS version 24 was used for statistical analysis (IBM Corporation, New York, NY, USA). Categorical data frequencies were examined using the Pearson Chi-Square and Fisher’s Exact tests. Visual inspection of histograms confirmed a non-Gaussian data distribution so that the Mann-Whitney U and Kruskal-Wallis tests were used to compare group distributions and Spearman’s Rho for correlations. In order to obtain predicted UIE values based on spot UIC concentrations, an estimation of 24 h urinary volume is required. Creatinine concentration serves as a surrogate for the state of concentration or dilution of the urine, varying inversely with urine volume. We used three different published equations based on age, weight, and height to calculate the predicted 24 h urinary creatinine excretion (PrCr): (1) The Tanaka equation [26]; (2) the Kawasaki equation [27]; and (3) an adapted Mage equation [28] (Table 1). The predicted 24 h creatinine excretion, calculated using each of these equations, was then used to determine the predicted 24 h iodine excretion [PrUIE] as follows: PrUIE = [(spot iodine (μg/L)/spot creatinine) × PrCr]. Differences between the measured UIE (24 h urinary volume (L) × aliquot of iodine from 24 h collection (μg/L)) and PrUIE were assessed using the non-parametric independent samples Mann-Whitney U test. To investigate whether spot UIC can be used to estimate mUIE, agreement between the predicted UIEs from spot urine samples using the different formulas and the measured UIE was assessed using Bland-Altman plots and assessment of limits of agreement (LOA). The LOA approach provides an informative analysis of reliability, including information about the magnitude of errors between methods. The 95% LOA represents a range of values within which, 95% of all differences between methods are expected to fall. Using the standard deviation (sd) of differences between methods, the 95% LOA were calculated for each of the three PrUIEs as mean agreement ±1.96 (sd diff). Both mUIE and PrUIE values were transformed to their natural logarithms (ln) before analyses because of the skewness in distributions. These are reported as the antilogarithm of the difference i.e. the geometric mean of the mUIE/PrUIE ratios and the antilogarithms of the LOA, which provide an interval within which 95% of the ratios lie [29]. For example, mean agreement of 100% suggests exact agreement, whereas mean agreement of 80% indicates that the PrUIE underestimates mUIE by 20%, on average. In the case of LOA values of 40–200%, this would suggest that 95% of PrUIE estimates are between 60 % underestimation and twofold overestimation, compared to mUIE value. 

Pr24hCr (mg/d) for women = 0.00163 3 (140 2 age (year)) 3 (weight (kg)^1.5^ 3 height (cm)^0.5^) 3 (1 + 0.18 3 A 3 (1.429–0.0198 3 BMI (kg/m^2^)), A median UIC (MUIC) of <100 μg/L indicates a population-level deficiency (there is no reference range for individuals) [34]. The EAR cut point approach, recommended by Zimmerman [35], was applied in order to assess the proportion of participants that would be considered to have an inadequate iodine status. This was applied to both the measured and predicted UIE values to provide information about potential bias in assessing the proportion of people with inadequate intakes of iodine if using the spot UIC values to estimate PrUIE. To convert urinary excretion values to estimated daily iodine intake (μg/day), both mUIE and PrUIE were divided by 0.92 to account for the 92% of dietary iodine that is absorbed [35]. The proportion of the population that had iodine intakes below the Estimated Average Requirement of 95 μg/day was compared across the measured and predicted values generated by the three equations. We further compared the proportion of subjects who would be considered iodine deficient (or insufficient iodine intake) based on the MUIC, predicted 24 h UIE (PrUIE), and measured 24 h UIE (mUIE) using respective cut-offs to determine whether MUIC over or underestimates iodine deficiency in a population. Iodine intake (µg/day) was also calculated using the IOM equation of ((UIC (μg/ L)/0.92) × (0.0009 L per h per kg × 24 × weight (kg))) [36] where 0.92 refers to 92% bioavailability and 0.0009 L per h per kg refers to the excreted urine volume from studies in children. 

## 3. Results

Characteristics of the study cohort by sex are shown in Table 2. More women than men were included and the median age was 52 (IQR 24) years, with 65% of the sample being older than 50 years. The Cohens Kappa statistic for inter-rater agreement between 24 h UIE and PrUIE using the Tanaka, Kawasaki and Mage equations indicated poor agreement (0.351, 0.324, and 0.309, respectively; all *p* < 0.001) [31] (Table 3). According to 24 h UIE, 41.1% of participants had an estimated daily iodine intake below the EAR, which differed significantly from estimates using PrUIE calculated using the Tanaka, Kawasaki, and Mage equations (39.3%, 41.3%, and 49.4%, respectively; all *p* < 0.05) (Table 4). Comparing only the subsample of subjects with PrUIE calculated from Mage equations (*n* = 428), the median PrUIE remained similar to the larger sample (*n* = 454), described in Table 4 (130 (139) and 123 (137) for the Tanaka and Kawasaki equations, respectively). The sensitivity of the equations to detect iodine intakes below the EAR of 95 μg/day was 59.9%, 60.4%, and 68.2%, for the Tanaka, Kawasaki. and Mage, respectively. Specificity to detect 24 h iodine intakes above the EAR of was 75%, 72%, and 63.6%, respectively.

The IOM weight-based equation [37], used to estimate the proportion of participants with dietary intakes below the EAR of 95 µg/day, identified only 34.7% of those categorised as such using measured 24 h UIE (X^2^ test; *p* < 0.0001).

**Figure 1:**
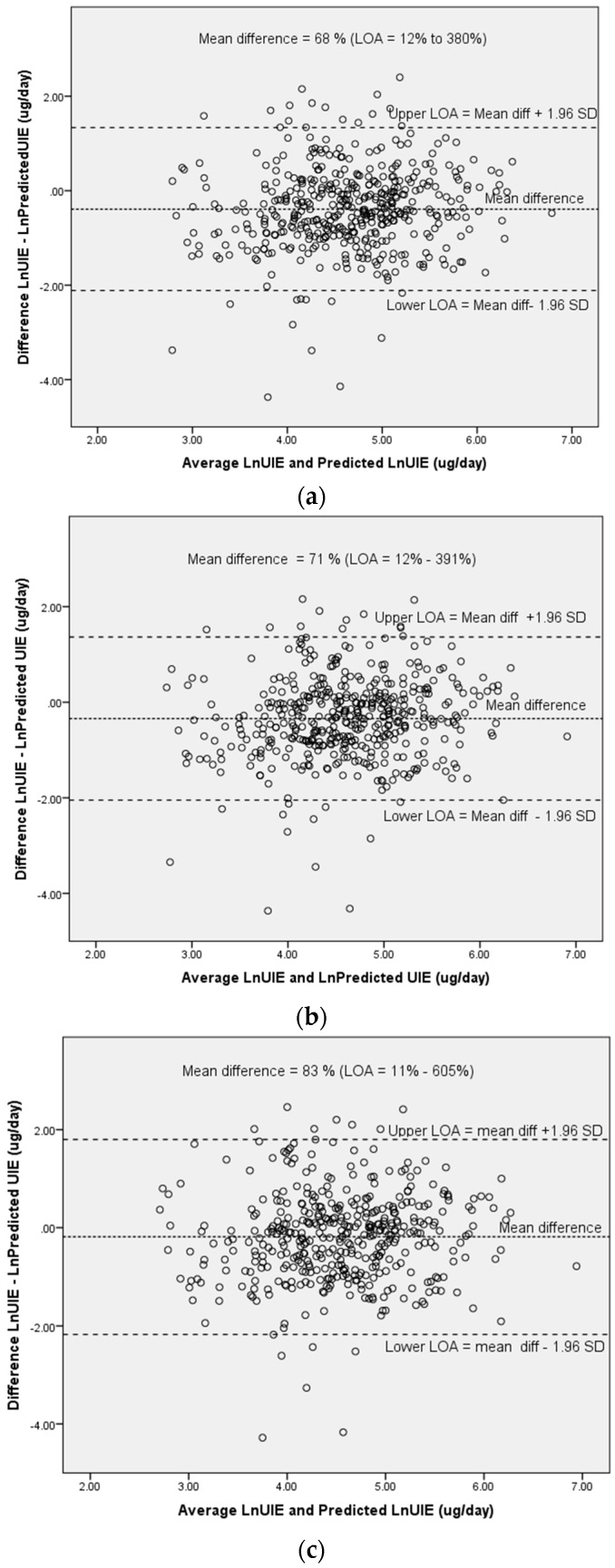
Bland Altman plots for the mean of natural logarithmic (Ln) transformed measured 24 h UIE and Ln transformed predicted UIE against the difference between ln transformed measured 24 h UIE and predicted UIE, using the following equations: (**a**) Tanaka; (**b**) Kawasaki; and (**c**) Mage equations; LOA = Limits of Agreement (Ln mean difference between measured and predicted UIE +/− 1.96 SD; shown as antilog or geometric mean, expressed as a ratio of difference between predicted UIE and measured 24 h UIE).

Bland Altman plots are shown in Figure 1a–c for natural logarithmic (ln) transformed values of predicted and measured UIE. Mean differences between values and limits of agreement (LOA) are presented as as the anti-log of the arithmetic mean of the ln-transformed values (i.e., the geometric mean). Thus, differences between PrUIE and mUIE were as follows: Tanaka: 68% (LOA 12–380%); Kawasaki: (71% (13–391%); and Mage: 83% (11–605%). The Mage equation (Figure 1c) had the widest LOA, but all three equations had unacceptably wide LOAs, indicating poor agreement with mUIE.

## 4. Discussion

The purpose of the current study was to assess the degree to which a spot urinary iodine concentration could be used as a proxy for measured 24 h urinary iodine excretion, for the purpose of assessing the adequacy of dietary iodine intake. The use of spot UIC values to indicate suboptimal iodine status (39% with MUIC <100 µg/L) provided a similar estimate to 24 h UIE (41% <EAR of 95 µg/day) at the group level. The use of spot UIC values in prediction equations to estimate 24 h UIE in this sample of South African adults provided a reasonable estimate of the prevalence of insufficient intakes below the EAR of 95 µg/day. For predicted 24 h UIE, based on spot UIC concentrations, all three prediction equations resulted in average under-estimations, ranging from 17% to 32 % for the different equations, with accompanying unacceptably wide limits of agreement. The difficulty of deciding on clinically relevant acceptable limits of agreement has previously been discussed by various authors [37,38]. In the case of iodine, for populations where intakes may be inadequate and where the consequences of inadequacy have serious impacts on health outcomes, such as in the case of pregnant women, more stringent cut-offs for determining acceptable limits of agreement may be warranted.

Our data suggests that spot UIC values provide an acceptable indication of population level deficiency, as compared to the EAR cutpoint method using measured 24 h UIE. Complex prediction equations applied to spot UIC values offered no greater accuracy and, rather, introduced major bias. Our findings are in contrast to those reported by Perrine et al. (2014) [39] in a study of younger healthy adults aged 18–39 years, in which UIC provided a reasonable estimate of 24-h UIE when prediction equations were used to determine 24-h urinary creatinine excretion.

Despite serious bias in predicted UIE, we did not find this bias to be systematic at higher levels of UIE. This is important in iodine replete populations that have been exposed to a well-functioning iodine fortification programme for some years and that may be at risk of potentially adverse excessive levels. A recent study of South African infant-mother pairs found that 21% of households consumed salt iodized above the upper level of 65 ppm and that the median UIC in the infants aged 2–4 months was more than three times higher than the WHO UIC threshold [40]. It is noteworthy that most recent estimates (2016) indicate that 10 countries have median urinary iodine concentration (MUIC) values considered to be in the excessive range (MUIC > 300µg/L) [4,5]. High iodine intakes are associated with iodine-induced hyperthyroidism and autoimmune thyroid disease [41,42], but there is a lack of consensus regarding the iodine intake that constitutes an adverse risk to health as indicated by a wide range in tolerable upper levels between countries. The WHO has expressed a cautious approach regarding upper levels, stating that a daily intake higher than 500 mg per day in pregnancy and lactation and more than 180 mg per day in children younger than two years is not necessary because it may, theoretically, be associated with impaired thyroid function [43].

As daily creatinine excretion is fairly constant at about one gram per day in healthy, well-nourished adults, it has been proposed that expression of UIC per gram of creatinine approximates the value in a 24-h collection and reduces variation due to hydration status [44]. However, malnourished populations with low protein intakes tend to have more variable daily creatinine excretion that is often lower than one gram per day [45]. In these settings, expressing the UIE as mg iodine/g creatinine may introduce greater, rather than less, variation. In our sample, dietary protein intake data was not available to test this hypothesis and the lack of reference values for UIC expressed as per gram of creatinine limits further interpretation. 

While the use of spot urine samples to monitor the iodine status of a population is widely accepted, there is potential for error in using the current MUIC reference cut-offs of <100 µg/L to indicate iodine deficiency in adults. The original MUIC reference cut-off values were determined based on a daily urinary volume of 1 L (as would be the case for school aged children), such that the UIC would approximate the UIE. However, in adults, who tend to have urine volumes that approximate 1.5 L/day [46], the UIC in µg/L is not equivalent to the 24 h UIE, expressed as µg/24 h. Based on the expected adult urine volume, UIC (µg/L) in spot samples could be expected to be about 60–65% of the amount excreted in 24 h [35]. Therefore, in adults, a UIE of 100 µg/24 h corresponds to a UIC of approximately 60–70 µg/L and this has been proposed by Zimmermann and Andersson (2012) [35] as being a more appropriate cut-off to indicate deficiency in adults. The Estimated Average Intake (EAR) value for iodine has been derived from balance studies and from studies measuring the daily iodine uptake, accumulation, and turnover in the thyroid gland using radioactive iodine in euthyroid adult subjects. These studies indicate that, to achieve iodine balance, the daily iodine intake (EAR) must be sufficient to enable the thyroid to turn over 95 mg iodine per day to maintain euthyroidism [36].

The large variation in a single spot urine iodine sample from day to day within individuals increases the spread of the distribution [16,47] so that it does not reflect the range of long-term or ‘usual’ iodine status around the median in a population. There are various available methods to reduce or remove the effects of measurement error due to the intra-individual variation that results from collecting a single-spot urine sample in population survey data. One method is to collect repeat samples over multiple days and average the data for each participant, but this substantially increases costs and logistics when conducting a national survey. Another method is to apply a correction factor to the distribution [47,48]. This requires estimation of a correction factor by collecting multiple samples from a representative subset of the survey population. Our group previously applied this method to three repeated UIC measures in a sample of healthy older Australians in the period prior to the introduction of mandatory iodine fortification [49]. After statistical adjustment for intra-individual variation, the proportion with UIC <50 μg/L reduced from 33% to 19%, while the proportion with UIC ≥100 μg/L changed from 21% to 17%, and the 95th centile for UIC decreased from 176 to 136 μg/L.

The EAR cut point method proposed by Zimmermann and Anderson (2012) [35] has been widely applied as an evaluation tool for nutrient intakes of groups [50,51] and to define the optimal fortification level of nutrients in foods [52]. The EAR cut-point method can be used to estimate the prevalence of iodine deficiency based on UIC distributions. The population distribution of UICs is typically skewed towards lower intakes, with a scattered tail of high intakes because of wide day-to-day variability in intakes. Assuming an adequate sample size of the group to account for inter-individual variation in the population distribution of UIC, it is possible to adjust the distribution to account for intra-individual variation using the National Cancer Institute or other similar statistical approaches [53,54]. This requires two or more repeated spot urine samples from the same individual in a subset of the study population in order to adjust the intake distribution closer to the mean. The Institute of Medicine suggest that daily iodine intake can be estimated using the weight-based equation: ((UIC (µg/L)/0.92) × (0.0009 L per h per kg × weight (kg))) [36]. In this equation, 0.92 refers to 92% bioavailability and 0.0009 L per h per kg refers to the excreted urine volume from studies in children. The estimated iodine intakes are adjusted for intra-individual variability and, thereafter, the proportion of individuals below the EAR (95 µg/day) can be ascertained to estimate the prevalence of iodine deficiency.

In our sample of adult South Africans intra-individual variability could not be assessed as only a single spot and 24 h urine sample was collected from each individual. Konig et al. (2011) [15] have reported a trend for higher intra-individual variation for spot UIC (38%) compared to measured 24 h urinary iodine excretion (32%) and this warrants further consideration. Other limitations include the sample population not being nationally representative as it was biased towards older adults aged more than 50 years, as well as the time of day for spot urine collection not being recorded and the lack of dietary intake data. Additionally, no information was collected on thyroid disorders nor on the use of thyroid medications in the SAGE sample. Therefore, we were unable to consider this in the selection criteria. It is, however, unlikely that many participants would have been diagnosed with thyroid disorders or taking medication for these conditions, since management of the condition on the continent is inadequate [55]. Strengths of the study design include a relatively large sample from all regions of the country, including inland, coastal, and mountainous regions. Furthermore, all analysis was conducted in one central laboratory, with quality control according to the CDC-EQUIP protocol (Ensuring the Quality of Iodine Procedures (EQUIP), Centers for Disease Control and Prevention) [21].

## 5. Conclusions

In a sample of adult South Africans, iodine status, assessed using median urinary iodine concentration at the group level, closely approximates urinary iodine excretion from 24 h urine collections. The use of complex prediction equations that incorporate spot UIC does not appear to offer additional accuracy in assessing population iodine deficiency. Continued use of the pragmatic collection of spot urine samples to assess population iodine status is supported.

## Figures and Tables

**Table 1:** Prediction equations used to estimate 24 h urinary creatinine excretion (PrCr).

Equation for Estimating Predicted 24 h Creatinine Excretion	Notes	Reference
Pr24hCr (mg/day) = (−2.04 × age (year)) + (14.89 × weight (kg)) + (16.14 × height (cm)) − 2244.45.	Developed in 591 Japanese adults aged 20–59 year	Tanaka, T.; Okamura, T.; Miura, K.; Kadowaki, T.; Ueshima, H.; Nakagawa, H.; Hasimoto, T. A simple method to estimate populational 24-hour urinary sodium and potassium excretion using a casual urine specimen. *J. Hum. Hypertens* **2002**, *16*, 97–103. [26]
Pr24hCr (mg/day) for men = (12.63 × age (year)) + (15.12 × weight (kg)) + (7.39 × height (cm)) – 79.9 Pr24hCr (mg/day) for women = (−4.72 × age (year)) + (8.58 × weight (kg)) + (5.09 × height (cm)) − 74.5	Equation for predicted 24-h urine creatinine excretion developed in a study of 256 male and 231 female participants aged 20–79 year [30] and validated in 20 male and 27 female Japanese and foreign (including 16 American) subjects.	Kawasaki, T.; Itoh, K.; Uezono, K.; Sasaki, H. A simple method for estimation of 24 h urinary sodium and potassium excretion from second morning voiding urine specimens in adults. *Clin. Exp. Pharmacol. Physiol*. **1993**, *20*, 7–14. [27]
Pr24hCr (mg/day) for men = 0.00179 × (140 − age (year)) – (weight (kg)^1.5^ × height (cm)^0.5^) × (1 + 0.18 × A × (1.366–0.0159 × BMI (kg/m^2^)) Pr24hCr (mg/day) for women = 0.00163 × (140 − age (year)) × (weight (kg)^1.5^ × height (cm)^0.5^) × (1 + 0.18 × A × (1.429–0.0198 × BMI (kg/m^2^)), where A is African American or black race = 1, other race = 0.	The Mage equation was developed to predict urine pesticide and chemical exposure with NHANES urine specimens. Equation for predicted 24-h urine creatinine excretion developed in a separate study [31] of 249 men in Canada with corrections based on the relative amounts of fat and muscle mass in women and differences in muscle mass by race and BMI.	Mage, D.T.; Allen, R.H.; Kdali, A. Creatinine corrections for estimating children’s and adult’s pesticide intake doses in equilibrium with urinary pesticide and creatinine concentrations. *J. Expo. Sci. Environ. Epidemiol.* **2008**, *18*, 360–368. [28] Huber, D.R.; Blount, B.C.; Mage, D.T.; Letkiewicz, F.J.; Kumar, A.; Allen, R.H. Estimating perchlorate exposure from food and tap water based on US biomonitoring and occurrence data. *J. Expo. Sci. Environ. Epidemiol*. **2011**, *21*, 395–407. [32]

Table adapted from Cogswell et al. (2013) [33].

**Table 2:** Characteristics of the study cohort by sex, WHO Study on global AGEing and adult health (SAGE) South Africa Wave 2 (2015).

	All *n* = 457	Men *n* = 109	Women *n* = 348	*p* Value
Age (years)	52 (24)	50 (23)	54 (23)	0.072
Aged over 50 years, *n* (%)	298 (65)	63 (58)	235 (68)	0.066
Ethnicity, *n* (%)				
Black African	315 (73)	77 (73)	238 (73)	0.248
Coloured, mixed race	70 (16)	16 (15)	54 (17)	
Indian	36 (8)	7 (7)	29 (9)	
White	10 (2)	5 (5)	5 (2)	
Rural, *n* (%)	131 (29)	31 (28)	100 (29)	0.926
Education (years)	9 (5)	10 (4)	8 (6)	0.001
Currently employed, *n* (%)	83 (31)	37 (51)	46 (23)	<0.001
BMI kg/m^2^	29.2 (9.1)	25.7 (7.3)	30.3 (9.3)	<0.001
Waist to height ratio, mean ± SD	0.58 ± 0.13	0.53 ± 0.11	0.60 ± 0.13	<0.001
Never used alcohol, *n* (%)	287 (81)	59 (63)	228 (88)	<0.001
Spot urinary cotinine (ng/mL)	19.1 (753)	19 (843)	19 (725)	0.867
Median UIC (µg/L)	130 (129)	149 (124)	121 (131)	0.102
UIC < 100 µg/L, *n* (%)	181 (39.5)	38 (34.9)	143 (41.0)	0.255
UIC < 50 µg/L, *n* (%)	70 (15.3)	14 (12.8)	56 (16.1)	0.450
Spot urinary iodine per creatinine (µg/g)	102 (103)	102 (106)	102 (99)	0.305
24-h urinary volume (mL/day)	1400 (1390)	1450 (1350)	1370 (1430)	0.929
24-h urinary iodine (mUIE) (µg/day)	124 (134)	137 (190)	119 (121)	0.010

All data is shown as median (IQR, interquartile range) unless otherwise indicated. Hypertensive categorised as BP ≥ 140/90 mmHg or previous diagnosis; Tobacco use/exposure identified by urinary cotinine analysis; BMI, body mass index; UIC, spot Urinary Iodine Concentration; mUIE, measured 24 h Urinary Iodine Excretion. Continuous median variables compared using Independent Samples Mann-Whitney U test and mean values with independent *t*-test; categorical variables compared using the Pearson Chi-Square and Fisher’s Exact Test.

**Table 3:** Agreement between measured (mUIE) and Predicted (PrUIE) urinary iodine excretion using prediction equations (*n*).

		mUIE †		
	Below EAR (<95 µg/day)	Above EAR (>=95 µg/day)	Total	*Kappa* Statistic *p* Value
Tanaka PrUIE				
Below EAR	112	67	179 (39.3%)	0.351
Above EAR	75	201	276 (60.7%)	<0.001
Total	187 (41.1%)	268 (58.9%)	455	
Kawasaki PrUIE				
Below EAR	113	75	188 (41.3%)	0.324
Above EAR	74	193	267 (58.7%)	<0.001
Total	187 (41.1%)	268 (58.9%)	455	
Mage PrUIE				
Below EAR	120	92	212 (49.4%)	0.309
Above EAR	56	161	217 (50.6%)	<0.001
Total	176 (41.0%)	253 (59.0%)	429	

† Daily iodine intake assumed as 24 h UIE (µg/day)/0.92 to account for bioavailability. PrUIE, Predicted Urinary Iodine Excretion (µg/day); EAR, Estimated Average Requirement (µg/day); mUIE, measured 24 h Urinary Iodine Excretion (µg/day).

**Table 4:** Difference between measured and predicted 24 h urinary iodine excretion (UIE), SAGE South Africa Wave 2 (2015).

Prediction Equation	*n*	Median (IQR)	Median (IQR) Difference †	Mann Whitney Test *p* Value	Spearman Correlation Coefficient *p* Value	EAR ‡ Below (%)
Mage equation (µg/day) mUIE PrUIE	428	124 (137) 105 (117)	17.8 (108)	0.000	0.402 0.000	49.5 *
Tanaka equation (µg/day) mUIE PrUIE	454	124 (136) 130 (136)	−3.2 (117)	0.399	0.413 0.000	39.3 *
Kawasaki equation (µg/day) mUIE PrUIE	454	124 (136) 122 (131)	4.1 (110)	0.443	0.425 0.000	41.3 *

PrUIE, Predicted Urinary Iodine Excretion (µg/day); mUIE, measured 24 h Urinary Iodine Excretion (µg/day). † mUIE minus PrUIE; ‡ EAR = 95 µg/day. Percentage below EAR is shown for each of the equations (X^2^ test compared to mUIE (41%); * *p* < 0.001). Mage n is lower as equation requires additional data on ethnicity (*n* = 26 with missing ethnicity data).

## Data Sharing Statement

The dataset is available on request and will form part of the data catalogue of the World Health Organization Study on Global AGEing and Adult Health (SAGE) [56].
